# Pseudorabies Virus Prevalence in Lung Samples of Hunted Wild Boars in Northwestern Greece

**DOI:** 10.3390/pathogens13110929

**Published:** 2024-10-25

**Authors:** Konstantinos Papageorgiou, Aikaterini Stoikou, Dimitrios K. Papadopoulos, Efpraxia Tsapouri-Kanoula, Ioannis A. Giantsis, Dimitrios Papadopoulos, Efthymia Stamelou, Marina Sofia, Charalambos Billinis, Chrysanthi Karapetsiou, Evanthia Petridou, Spyridon K. Kritas

**Affiliations:** 1 Laboratory of Microbiology and Infectious Diseases, School of Veterinary Medicine, Faculty of Health Sciences, Aristotle University of Thessaloniki, 54124 Thessaloniki, Greece; kstoikou@gmail.com (A.S.); eftsapourivet@yahoo.gr (E.T.-K.); dpapvet@hotmail.com (D.P.); efistamel@hotmail.com (E.S.); epetri@vet.auth.gr (E.P.); skritas@vet.auth.gr (S.K.K.); 2 Department of Animal Science, Faculty of Agricultural, Forestry and Natural Environment, Aristotle University of Thessaloniki, 54124 Thessaloniki, Greece; dkpapado@bio.auth.gr (D.Κ.P.); igiants@agro.auth.gr (I.A.G.); 3 Laboratory of Microbiology and Parasitology, Faculty of Veterinary Medicine, University of Thessaly, 43100 Karditsa, Greece; msofia@vet.uth.gr (M.S.); billinis@uth.gr (C.B.); 4 Veterinary Directorate, Region of Epiros, 45332 Ioannina, Greece; ch.karapetsiou@php.gov.gr

**Keywords:** pseudorabies, wild boar, pigs

## Abstract

Aujeszky’s Disease, caused by the pseudorabies virus (PRV), is an acute, often fatal disease affecting mainly pigs and incidentally other animals. While eradicated in several countries, PRV persists in wild boar populations, posing a risk to domestic pigs. This study investigates PRV prevalence in wild boars in the region of Epirus, located in the northwest of Greece. During the 2021–2022 hunting season, 110 lung samples from hunted wild boars were collected and analyzed for PRV DNA and cytopathic effects in cell cultures. PRV DNA was detected in 19 samples (17.3%), 18 of which exhibiting cytopathic effects, allowing for virus titer determination. Notably, in one sample, PRV DNA was detected without a cytopathic effect. These findings underscore the continued presence of PRV in Greek wild boars, highlighting the need for ongoing monitoring to prevent transmission to domestic pigs and other animals.

## 1. Introduction

Aujeszky’s Disease is an acute and often fatal disease, primarily affecting pigs and occasionally impacting other domestic and wild animals, which causes significant economic losses to the swine industry in affected regions [1,2]. Initially described in 1813 in cattle exhibiting extreme pruritus, the disease was termed “mad itch”. The name “pseudorabies” was adopted due to its clinical similarity to rabies [3,4]. The Hungarian veterinarian Aladar Aujeszky, after whom the disease is currently named, first described and reproduced it in 1902, demonstrating that the etiological agent was filterable, indicating that it was not a bacterium [5].

The pseudorabies virus is classified in the genus *Varicellovirus*, subfamily Alphaherpesvirinae, family Herpesviridae [6]. While pigs are the primary natural hosts, PRV can infect various animals, including carnivores, ruminants, and rodents [7]. PRV is globally distributed, found in parts of Europe, Southeast Asia, and America. In Europe, pseudorabies has been eradicated from domesticated pigs in countries such as Austria, Cyprus, the Czech Republic, Denmark, Finland, France, Germany, Hungary, Luxembourg, the Netherlands, Sweden, Switzerland, Norway, and the United Kingdom. However, it remains endemic in eastern and southeastern Europe [2,8,9]. Canada, the United States, and New Zealand are also free of PRV [10]. The successful eradication in domestic pigs is attributed to national programs that included large-scale vaccination with gE-deleted vaccines, part of the DIVA (Differentiating Infected from Vaccinated Animals) strategy [2,11]. Although PRV has been eradicated in many countries, it continues to circulate in wild boar populations [12,13,14,15,16,17]. Therefore, wild boars should be considered potential PRV sources of infection for domestic pigs.

Greece is among the countries where the disease remains enzootic. A serological study from 1969 found that 20.8% of domestic pig samples from various Greek regions were positive for PRV antibodies [18]. In 2010, Papageorgiou et al. conducted an epidemiological study to investigate PRV antibody presence in Greek domestic pigs [19]. Pig herds were selected based on geographical criteria to provide representative data from across Greece. The study revealed that 28.6% of the examined pig farms tested positive for PRV antibodies. Despite the presence of PRV in Greece’s domestic pig population, there is no centralized plan for eradicating the disease. While pig farmers commonly vaccinate their herds using attenuated vaccines, they do not implement the DIVA strategy, which is essential for disease eradication efforts.

Wild boars are considered reservoirs for several pathogens, including PRV and the African Swine Fever Virus (ASFV) [12,20]. In Greece, studies from 2015 reported the presence of PRV antibodies in 32% to 35% of wild boar populations [21,22]. Moreover, Greece was considered free of ASF until 2019 when an outbreak occurred in a backyard pig farm near the Bulgarian border, in which country ASF is enzootic. Given the significant role of wild boars in virus transmission, including ASF, the Greek authorities, in cooperation with hunting associations, began collecting tissues from hunted wild boars to investigate ASFV presence and prevent its transmission to domestic pig herds.

Additionally, encephalitis cases in hunting dogs that had contact with wild boar carcasses in the Epirus region (northwestern Greece) prompted a collaboration between our laboratory and local authorities. The isolation of PRV from the brain samples of these dogs underscored the need to further investigate the virus’s presence in wild boars. Consequently, this study investigated the prevalence of PRV in the lungs of hunted wild boars, in collaboration with the relevant authority in the Epirus region.

## 2. Materials and Methods

### 2.1. Study Area and Sampling

Our study was conducted in the Epirus region, located in the northwestern part of Greece. Epirus is characterized by its mountainous terrain, predominantly covered by forests. Over the past four years, the estimated population of wild boars in the country has shown a significant increase, rising from approximately 100,000 in 2020 to 400,000 in 2024 (personal communication with veterinary authority officers). According to data provided by hunting associations, an estimated 9000 wild boars are hunted annually in Greece.

During the 2021–2022 hunting season (20 August 2021 to 28 February 2022), a total of 110 tissue samples from hunted wild boars were collected by hunters from the Epirus region. These samples were transported in isothermal boxes, maintaining temperatures between 5 and 8 °C, to local authorities on the same day of collection. Subsequently, the samples were stored in a freezer at −80 °C. Upon completion of sampling, 110 lung samples were sent to the Laboratory of Microbiology and Infectious Diseases, School of Veterinary Medicine, Aristotle University of Thessaloniki. The samples arrived at the laboratory on the same day in isothermal boxes with temperatures around −20 °C and were tested the following day for the presence of PRV. As the wild boars were shot by hunters during the annual hunting season and not for the purpose of this study, no ethical permit was required for the present survey.

### 2.2. Virus Quantitation and PCR

For virus quantitation, 20% sample suspensions were prepared in PBS (Thermo Fischer Scientific, Waltham, MA, USA) and centrifuged at 3000 rpm for 20 min at 4 °C. Subsequently, 0.05 mL of the supernatant was inoculated onto each of four wells containing confluent ST cells (ATCC CRL-1746), which were kindly provided by the Laboratory of Virology, Faculty of Veterinary Medicine, University of Ghent, Belgium. The ST cells were cultured in a medium based on MEM + GlutaMAX (Gibco, New York, NY, USA) supplemented with 5% fetal bovine serum (Gibco, New York, NY, USA), 1% penicillin-streptomycin (Gibco, New York, NY, USA), and 0.5% gentamycin, and incubated at 37 °C under 5% CO_2_. The cell cultures were monitored for cytopathic effects over a five-day period post-inoculation. For quantitation, 10-fold serial dilutions of the initial suspension were performed, and end-point titers were determined using the Reed–Muench method.

To investigate the presence of viral DNA in the lung samples, a conventional PCR protocol was employed to amplify a fragment of the gE gene. DNA was extracted from the lungs using the QIAamp DNA Mini Kit (Qiagen, Hilden, Germany), following the manufacturer’s instructions. The concentration and purity of the extracted DNA were assessed using an Eppendorf BioSpectrophotometer (Eppendorf, Wien, Austria). The primer sequences used were as follows:

Forward primer (gE-nF): 5-CCGCGGGCCGTGTTCTTTGT-3;

Reverse primer (gE-nR): 5-CGTGGCCGTTGTGGGTCAT-3. 

These primers amplify a 493 bp part of the gE glycoprotein of the virus [23]. PCR reactions were set up using the KAPA Taq HotStart PCR Kit (Sigma-Aldrich, St. Louis, MO, USA). Each 20 μL reaction mixture contained 4 μL of 5× KAPA Taq HotStart buffer, 2 μL of MgCl2, 0.4 μL of a 10 mM dNTP mix, 0.3 μL of each primer (10 mM), 0.2 μL of 5 U/μL Taq polymerase, 2 μL of sample DNA, and 10.8 μL of nuclease-free water. Amplification was performed in an ABI Veriti 96 Thermal Cycler (Applied Biosystems, Foster City, CA, USA). The PCR products were resolved on a 2.0% agarose gel via electrophoresis under standard conditions and stained with ethidium bromide (2 μL/mL). 

## 3. Results

Out of the 110 samples tested, 19 (17.3%) were positive for PRV DNA. The amplified part was totally identical with the reference strain sequence with GenBank accession number AY249861. Additionally, a cytopathic effect was observed in cell cultures from 18 of the 110 lung samples, allowing for the determination of the relevant virus titer. Table 1 shows the results of virus titration and PRV molecular confirmation. 

According to the results presented in Table 1, the viral DNA was detected in all samples where PRV caused a cytopathic effect. In one sample, the presence of the virus DNA was confirmed without any observed cytopathic effect.

## 4. Discussion

The wild boar is the most widely distributed species of the Old World Suidae family and has the broadest geographic range of any terrestrial mammal [2]. These free-living swine are known reservoirs for numerous pathogens including bacteria, viruses, and parasites, which can be transmitted to domestic pigs and other animal species [12]. Notably, wild boars play a significant role as reservoirs for PRV, with the virus being more prevalent and widespread in these than previously understood. Numerous studies across Europe have documented PRV in wild boar populations, revealing a higher prevalence of PRV antibodies in the Mediterranean countries compared to Central Europe and Scandinavia [14]. In Italy, the seroprevalence of PRV in wild boars ranges from 7.9% in the northern part of the country to 23.85% in southern regions [24,25,26,27]. In Germany, the seroprevalence is 12.09% [28] while Slovenia and Spain have reported the presence of PRV antibodies in 31% and 36.63% of the tested wild boars, respectively [29,30]. Conversely, in Switzerland, the Netherlands, and Sweden, PRV is present in up to 4% of tested serum samples [31,32,33,34]. The high prevalence of PRV in wild boar populations increases the risk of virus transmission, not only to domestic pigs but also to other animals. This situation is particularly concerning for this region of Greece, which has a high wild boar population. Given the lack of studies from this area on PRV presence in other animal species, further investigation is crucial to prevent the potential underreporting of the disease in these species.

Our study identified the presence of PRV DNA in 17.3% (19 out of 110) of lung samples from hunted wild boars in the Epirus region. In addition, the virus titer was detected in 16.4% (18 out of 110) of the samples, a fact that can be attributed to the higher sensitivity of PCR compared to the virus isolation method [35,36]. The PRV prevalence observed in our study using the molecular technique is lower compared to the results of other Greek studies, which reported PRV antibodies in 32% and 35% of serum samples from hunted wild boars. However, in our study, we confirmed the presence of viral DNA whereas we also calculated the viral titers, indicating active infection in the lung samples. In contrast, PRV antibodies in serum samples may reflect past infections, which could explain the lower percentage of positive samples in our study.

In wild boars, PRV infection typically occurs without clinical symptoms, except in younger animals, where nervous system symptoms can occasionally be observed [37]. Compared to domestic pigs, very young wild swine appear to have greater resistance to the virus [38]. Unfortunately, our study lacked information about the symptoms of the animals before they were shot. Nonetheless, hunters reported that in some cases, the wild boars moved more slowly than expected, appeared dizzy, and showed poor awareness of environmental conditions. This study was primarily designed due to reported cases of encephalitis in hunting dogs that had close contact with hunted wild boars. Direct contact with PRV-infected wild swine poses a potential risk of transmission to carnivores. Several studies have reported the isolation of PRV from hunting dogs after contact with wild boar carcasses. In France, from 1997 to 2004, 20 hunting dogs died from PRV infection originating from wild boars [39]. In Germany, from 1995 to 2022, PRV was reported in 35 hunting dogs [40]. Furthermore, numerous studies across Europe highlight the significant role of PRV in hunting dogs that have close contact with wild boars [41,42,43,44]. The clinical outcome in PRV-infected dogs is characterized by neurological signs, with death occurring 6 to 96 h after the onset of clinical symptoms [45]. This observation aligns with data from the Greek authorities, who received reports from veterinarians about cases of encephalitis in hunting dogs following contact with wild boars.

## 5. Conclusions

Our study underscores the ongoing presence and potential risks associated with PRV in wild boar populations in the Epirus region of Greece. The detection of PRV DNA in 17.3% of lung samples from hunted wild boars indicates active infections, despite the lower prevalence compared to serological studies in Greece. This discrepancy may be attributed to the detection of viral DNA, reflecting active infections, versus antibodies, which may indicate past exposures.

The significant role of wild boars as reservoirs for PRV poses a continuous threat to domestic pigs and other animals, including hunting dogs, as evidenced by the reported cases of encephalitis. These findings highlight the importance of ongoing surveillance and control measures to mitigate the spread of PRV from wild boar populations to domestic animals. Collaboration between veterinary authorities, hunting associations, and research institutions is crucial for effectively monitoring and managing this disease.

## Figures and Tables

**Table 1:** Results of virus titration and DNA detection from wild boar lung samples in the region of Epirus, northwestern Greece.

	Sample	Virus Titer (log TCID50/g Tissue)	PRV DNA Detection
1	ASF-A-24	4	Detected
2	ASF-A-40	4.33	Detected
3	ASF-A-42	3.33	Detected
4	ASF-A-77	3.67	Detected
5	ASF-A-108	3	Detected
6	ASF-A-117	3.67	Detected
7	ASF-A-154	4	Detected
8	ASF-A-158	4	Detected
9	ASF-A-179	2.75	Detected
10	ASF-A-186	3.33	Detected
11	ASF-A-188	2.75	Detected
12	ASF-A-220	3.67	Detected
13	ASF-A-221	3.67	Detected
14	ASF-A-226	3.23	Detected
15	ASF-A-229	2.75	Detected
16	ASF-A-230	2.75	Detected
17	ASF-A-233	3.5	Detected
18	ASF-A-245	3.83	Detected
19	ASF-A-252	-	Detected

## Data Availability

Dataset available on request from the authors.
